# More pressure, more release? When compulsory care fails: an autonomy-supportive treatment policy as an alternative to failed compulsory care in inpatient care for youth with anorexia nervosa

**DOI:** 10.3389/fpsyt.2026.1797306

**Published:** 2026-05-12

**Authors:** Lisanne L. Stone, Manoek Albert, Carmen V. Voogt, Ella van den Berg, Esther bij de Vaate, Louise Schepman, Liesbeth Hoekstra, Pierre C. M. Herpers, Ciska Brakenhoff

**Affiliations:** 1 Developmental Psychology, Tilburg University, Tilburg, Netherlands; 2 Intensief Behandelcentrum Jeugd, Karakter, Ede, Netherlands; 3 Radboud University, Behavioural Science Institute, Nijmegen, Netherlands; 4 Geestelijke GezondheidsZorg (GGZ) Oost Brabant, Boekel, Netherlands; 5 Independent researcher, Lausanne, Switzerland

**Keywords:** anorexia nervosa, autonomy-support, coercion, compulsory care, systemic

## Abstract

**Introduction:**

Anorexia nervosa (AN) in young people is, in its most severe form, a life-threatening problem for which compulsory care may be indicated. In The Netherlands, this compulsory care takes place in closed crisis departments for child and adolescent psychiatry. At the same time, the provision of compulsory care within the context of a closed ward has limitations and unintended adverse effects and is colored by ethical dilemmas. Because of the scenario described above, an alternative treatment policy, which can be embedded systemically within relationality and ethically within a care framework, is proposed and is aimed at promoting autonomy.

**Methods:**

This study consists of two parts, where in part one both policies are described and in part two qualitative interviews with two youths were held.

**Results:**

The results indicate that the youths’ experiences point to reflections on recovery, intrusiveness, traumatization, and resistance regarding the coercive elements within compulsory care. Finally, recommendations on how to professionalize compulsory care, if any, are given by youths with severe AN.

**Discussion:**

The practical and clinical relevance of our results are that an alternative to compulsory care is possible and feasible. The scientific implications are that treatment rationales and treatment policies in compulsory care for AN are dominantly rooted in positivistic, linear modes of thinking. In a mixed-methods perspective, we argue how positivistic and constructionist approaches can be integrated to improve the overall quality of care for youths with severe AN.

## Introduction

First-person experiences of treatment for anorexia nervosa (AN) in the context of compulsory care have been described as life-saving (1) and helping (2) while simultaneously experienced as traumatizing (1) and associated with feelings of being hunted or assaulted (2). Notably, the experiences of compulsory care are often shielded to those not directly providing or receiving such treatment given that the treatment procedures take place in closed psychiatric wards. Therefore, studying this context from within (e.g., emic perspective), that is by treatment providers and receivers, and from without (e.g., etic perspective), that is by researchers who carefully listen to treatment providers and receivers (cf 3), offers a unique opportunity for formulating fresh perspectives. Moreover, given the interdisciplinary and localized practice that compulsory care for AN constitutes, studying this context fits with a pluralistic (4) and dynamic systems perspective (5)—that is, in the Dutch context of compulsory care for youth with severe AN, the medical, psychiatric, legislative, municipal, psychological, systemic, nursing, sociotherapeutic, and ethical disciplines collaborate with each other. Such collaboration may be dubbed a complex system, or, in keeping with a dynamic systems perspective, conceptualized of as an ecosystem (6). Central to all disciplines or stakeholders in such a system is the *intention* to provide good care for people with severe AN. As has been argued (7), prioritizing voices of stakeholders in answering the many and difficult ethical questions in the debate on what good care is for youth with severe AN is needed, especially so as there is a group of young people for whom a compulsory care policy is not effective. Therefore, this two-part study aims to (1) describe and critically reflect, from both theoretical and clinical perspectives, on an autonomy-supportive treatment policy as an alternative to compulsory care (i.e., treatment-as-usual) for youth with severe AN and (2) illustrate how adolescents with severe AN themselves experience both treatment approaches in a qualitative study, explicitly highlighting their voices as key stakeholders. We start by contextualizing the current treatment landscape in child and youth psychiatry in The Netherlands, reviewing scientific positivistic evidence, and then contextualizing such scientific evidence by reviewing evidence following a constructivist scientific paradigm.

**Figure 1:**
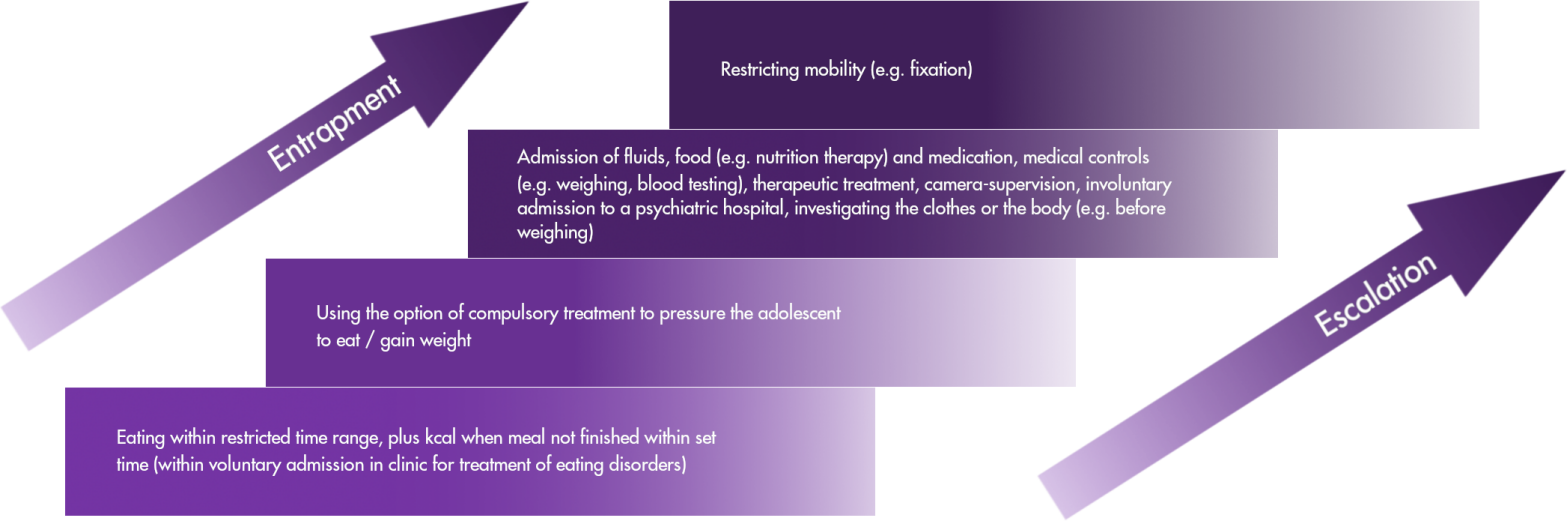
Escalation ladder depicting the development of coerciveness in care for anorexia nervosa.

To understand how compulsory care and its coercive practices in inpatient settings are situated within the broader spectrum of treatment options for AN, it may be helpful to imagine coercion as moving on an escalation ladder (8). This metaphor is specific to the Dutch situation, as coercive practices vary greatly across cultures and depend on local legislative practices (9). Importantly, the explanatory models of AN influence treatment practices with the as “egosyntonic” described nature of AN, where the illness is viewed as consistent with one’s self-image and own values rather than alien and distressing, helping to explain why treatment motivation can be difficult to establish (10) and why compulsory care is sometimes considered necessary. In first-line recovery-oriented treatments, such as family-based treatment (FBT; 11, 12) and cognitive behavioral therapy (CBT; 13), the focus is on collaboration with parents, cognitive restructuring, and changing unhealthy behavior patterns in order to restore a healthy weight. At first glance, coercion does not play any role in these treatments. However, parents have disclosed that they feel that their role in FBT is reduced to a warden to their child, as parents are made responsible for ensuring eating, weight gain, and the prevention of high-risk behaviors (14). In practice, this means parents supervise their children strictly during and following mealtimes. Instructions to finish a meal within a restricted time range and adding more kilocalories when the meal is not finished within this time (“plus kcal”) are also common practices in first-line recovery-oriented treatments and can be dubbed coercive as such, depicted at the bottom-end of the escalation ladder (**Figure 1**). In accordance with established clinical guidelines, when first-line recovery-oriented treatments for AN fail to yield adequate outcomes, families are typically offered a stepped-care intensification. These options include systemic therapy, pharmacological treatment, day-treatment programs involving a multidisciplinary team (e.g., dietitian, psychologist, psychiatrist), specialized inpatient treatment for eating disorders (EDs), and, in severe cases, compulsory treatment (15, 16). However, as Birch et al. (17) emphasize, such escalated approaches may not always align with the patients’ values or lead to sustained recovery, particularly in severe cases. In recognition of these limitations and drawing on the perspectives of both clinicians and service users (18), a growing paradigm shift is emerging toward harm-reduction models. These approaches, as outlined by Williams et al. (19), prioritize quality of life, patient autonomy, and collaborative care planning over traditional goals such as full weight restoration.

The legislated part of coercion in treatment (or compulsory care) usually starts, in The Netherlands, when adolescents^1^ refuse admission to a clinic while they are in danger to life, when their voluntary admission to a clinic for EDs leads to severe deterioration, or sometimes when they did not receive any kind of treatment yet but their weight has plummeted such that immediate specialized inpatient care is deemed necessary. In such cases, the Dutch situation is that a legal authorization for compulsory care is requested by an independent psychiatrist or clinical psychologist which is motivated by three guiding principles centered on proportionality, subsidiarity, and effectiveness and intended to protect the interest and health of the client. If deemed justified, the legal authorization is then issued by a judge (20). Usually, compulsory care is provided within youth psychiatric hospitals. Examples of coercive elements within compulsory care are administration of fluids, food (e.g., nutrition therapy) and medication, medical controls (e.g., weighing, blood testing), therapeutic treatment, camera supervision, involuntary admission to a psychiatric hospital, investigating the clothes or the body (e.g., before weighing), or restricting mobility (e.g., fixation). These practices are at the top-end of the escalation ladder. Also at the top end, increasing feelings of worry and powerlessness in adolescents with severe AN, parents, and the treatment team together with increasing escalation can be conceptualized as an entrapment dynamic.

While one might expect rigorous scientific evidence to underlie these coercive practices, the available evidence for the conventional practice of providing nutrition therapy and applying coercion to youth with severe AN is deemed weak (21, 22). Zooming in on specialized, often coercive, treatment settings for severe AN, a recent systematic review and meta-analysis reported that compulsory treatment yields similar outcomes to voluntary treatment in terms of BMI, illness duration, and mortality, albeit with 3 weeks longer hospitalizations for the compulsory-treated patients (23). It is important to note that, among the nine studies included in this systematic review and meta-analysis, only one specifically focused on a youth population (aged 16). Surprisingly, the authors conclude that “as the outcomes between compulsory- and voluntary-treated patients did not differ, we can assume that there are no beneficial neither detrimental effects in the two types of treatment”, thereby ignoring the impact of longer hospitalization, let alone the well-established impact of coercion on the person, their families, and professionals in the short and long term, respectively (24–30) Descending the escalation ladder, despite the multitude of research studies, treatment outcomes for people with AN have improved little during the last seven decades (22, 31–33). A network meta-analysis into the efficacy and acceptability of psychological interventions for adult outpatients with AN (34) shows that no reliable evidence supports the clear superiority or inferiority of the specific treatments that are recommended by clinical guidelines internationally.

When practitioners are confronted with a person presenting with critically low body weight and associated life-threatening somatic parameters, they often feel obliged to intervene to preserve life, particularly when the person is young. However, such intervention may occur despite the person’s explicit refusal of treatment. This dilemma exemplifies the characterization of AN as a “wicked problem”, a socially embedded issue perceived as complex, unpredictable, and lacking a clear or universally accepted solution (35). The lack of treatment motivation and refusal of treatment can be explained by an important explanatory model of AN—namely, the hypothesized egosyntonic nature of AN—which clashes with the severity that AN can present, where intervention and coercion can be deemed necessary to save a life. Qualitative research has shown that some young people with AN feel unheard within the context of compulsory care and, in certain cases, experience such interventions as traumatic (1, 2). The common conceptualization of recovery in AN—as predominantly physical recovery—within the biomedical positivistic context (36–39) appears to be limited and is perceived by young people with AN as unhelpful, although they also understand that the focus on physical recovery aiming at BMI normalization (which currently is acknowledged as being far from a satisfactory measure of illness severity; see 23) can be lifesaving (1, 40). However, while physical recovery and medical stabilization constitutes a necessary component of overall recovery in AN, it is not sufficient in isolation; comprehensive recovery should be conceptualized as a multidimensional process encompassing psychological, behavioral, and social domains (17, 18). Qualitative research shows that connecting with the person and their identity beyond the AN and continuing to offer hope are helpful (41). In the ambivalent process of recovery, helping to focus on values is essential (42). However, within the context of compulsory care, this remains a complex and challenging issue for the admitted youths, their parents, and the clinicians involved.

Qualitative research can be situated more broadly within social constructivism where it is assumed that reality is what we construct it to be within relational transactions (43–45). As such, social constructivism acknowledges the complexities surrounding the compulsory care for youth with AN, where ethical dilemmas regarding relational themes (e.g., who decides about the continuation of treatment, who cannot decide about the continuation of treatment, and why? Who is affected by the decision to continue compulsory care and how? What does the *intention* of providing good care mean in the context of compulsory care to youth and to parents? How can caregivers be assured that they are helping the person? How can the person perceive the intervention as an expression of care)? are commonplace. Importantly, these ethical dilemmas unfold within interpersonal relationships (i.e., relational transactions). Given the potential pitfalls of compulsory care, an alternative autonomy-supportive treatment policy was developed, which can be embedded in a systemic perspective wherein complexity and relationality is assumed.

“The pathology is to want control, not that you ever get it, because of course you never do.”

This quote by the founding father of systems thinking Gregory Bateson (6) alludes to the hypothesized internal process so often described as central to AN. Moreover, it resonates with the dynamic playing out in inpatient care settings where people with AN are admitted when in life-threatening danger. The hypothesis on how the internal psychological dynamic of attempting to gain control clashes with coercive practices in inpatient care settings for youth with severe AN guided our thinking about treatment policies. This study describes, investigates, and critically reflects, from both theoretical and clinical perspectives, on an autonomy-supportive treatment policy as an alternative following ineffective compulsory care (i.e., treatment as usual) for adolescents with severe AN. Furthermore, it investigates how adolescents with severe AN themselves experience both treatment approaches, explicitly foregrounding their perspectives as key stakeholders. First, we outline the treatment-as-usual policy within an inpatient care setting. Subsequently, we describe the development of an autonomy-supportive treatment policy, grounded in both professional consensus and clinical practice, followed by a qualitative analysis of experiential reflections provided by two adolescents with severe AN.

## Methods

### Design

The study consisted of two parts, namely: (1) describing the treatment policies and (2) collecting idiosyncratic narratives analogous to a case study. The treatment policies for part 1 of our study were compared by using a conceptual interdisciplinary analysis. Regarding part 2 of our study, the experiences of adolescents with severe AN were explored through a case study approach combined with a qualitative analytic framework, enabling in-depth examination of dynamic intrapersonal processes (46, 47). To facilitate open exploration, a qualitative research design was chosen, drawing on idiosyncratic narratives and reflective interview questions. Within the author team, ethical issues were addressed through the use of reflective team methods. The themes of discussion among team members were whether it would be suitable for youths to be contacted for the study and who of the team members would be most appropriate for seeking contact with youths. For the nurses in our author team, some personal contact remained with youths following discharge from the clinic. In such cases, it was deemed logical that the nurse would contact the youths. Ethical issues were resolved by prioritizing autonomy for the youths to choose whether to participate in this study and to acknowledge that our role was different than being an active caretaking professional where dependency on the caretaker would play a role in the relationship.

### Participants

The participants were recruited from a specialist center for child and youth psychiatry in The Netherlands. The inclusion criteria for study participation were (1) experiencing compulsory care in an inpatient clinic during adolescence for AN, (2) perception of currently being in the process of recovery, (3) being able to provide answers to interview questions in Dutch, and (4) providing active informed consent. The exclusion criteria were (1) intellectual disability (IQ <70) and (2) active psychosis. Three persons initially consented to participate; however, one subsequently withdrew, resulting in a final sample of two participants.

### Procedure

Regarding part 1 of our study, the procedure was to describe the treatment policies. The second author described the treatment policies, with EbdV and LS providing feedback on the texts and LS contextualizing the texts further by interpreting these from a social constructivist and care ethics perspective.

Regarding part 2 of our study, the procedure included contacting the participants by email, explaining the goal of the study, and asking for written active informed consent. Following receipt of consent, an idiosyncratic narrative was developed based on the psychiatric case file and subsequently shared with the participant in written form via email. The participant was invited to provide feedback on the narrative (i.e., member check; cf 3) and to give consent for publication. Following revisions based on this feedback and receipt of consent, the narratives were finalized by the first author. Subsequently, the participants were asked two reflective questions, namely: (1) please provide a reflection on coercive elements in your treatment, how these elements were helpful to you or not, and how they have influenced your recovery and (2) please provide a reflection on autonomy-supportive treatment, how elements in this treatment were helpful to you or not, and how they have influenced your recovery by email. They were given the option to respond either in writing or through a phone call, with phone interviews lasting about 30 min. When the participants chose phone call, the first author recorded notes in a Word document during the conversation. A summary of the statements provided during the phone call or via email was written up and presented to the participants for consent. Furthermore, the participants were invited to be included as co-authors via email, with the goal of equal collaboration in mind. One of the participants chose to discuss this matter on the phone with the first author and then decided to agree to this; the other chose to remain anonymous.

### Data analysis

Regarding part 1 of this study, the databases Web of Science and PsycInfo were searched by using the keywords “coercion”, “compulsory care”, “(care) ethics”, and “anorexia nervosa” in order to conduct the conceptual interdisciplinary analysis. The results from the literature search were incorporated in a conceptual analysis of the treatment policies as presented below.

As to part 2 of this study, reflexive thematic analysis was used as a method for making meaning from qualitative data (3), where both the role of the researcher in the process of meaning making is recognized and the iterative process of identifying elements of the data and determining themes is incorporated. Reporting was guided by the Standards for Reporting Qualitative Research (48). Codes and subsequently themes were identified by LS and discussed with CV, conforming to the notion that a single coder is considered good practice in reflexive thematic analysis (3). Given our small sample size and limited data, no specific software program was used for data analysis. The interviews were not recorded given their short length, deeming recording and transcribing unnecessarily complicating to the study. Data were analyzed using a social constructionist lens, with recognition of the power difference between the researchers, clinicians, and youths.

### Reflexivity statement

The research team includes people from both scholarly and clinical backgrounds, with all members coming from Global North countries. Some of us benefit from white privilege and thin privilege. The team comprises a mix between academically and vocationally trained persons. Some of us have lived experience with EDs, which helped inform the study’s design and analysis. Our backgrounds and lived experience inevitably shape our research and interpretations, which is why it is important to name this (49). Rather than presuming objectivity, naming our subjectivities and considering our biases are part of effectively and ethically doing research, as these allow us to contextualize and enrich the research process (50). Given the overlap in treatment and research team (excluding CV and CB), we reflect on how our treatment positions may have influenced our researcher perspective. First, witnessing harmful and non-effective effects of coercion may have influenced our research questions and drawing of conclusions more favorably toward the autonomy-supportive direction. Second, given the fact that we have been colleagues for over 5 years may have influenced our stance toward the autonomy-supportive policy, favoring it because it has been developed within our team by trusted and valued colleagues. Given the former and the fact that we have only been able to interview two informants, the results of the current study are considered tentative and should be interpreted accordingly.

## Results

### Part 1: Description and development of treatment policies within inpatient care for youth with AN

#### Treatment-as-usual policy: compulsory care

The following is based on the interdisciplinary perspective of psychiatrists, psychologists, nurses, sociotherapists, and ethicists. The treatment-as-usual policy means that the responsibility with regard to eating is shifted completely to parents or to clinicians by means of the use of compulsory care under the Law on Compulsory Mental Health Care (abbreviated in Dutch as WvGGZ). This shift of responsibility from the young person with severe AN to parents/clinicians is based on the idea that the young person is deemed unable (or no longer able) to take healthy decisions with regard to eating (16). In severe AN, where admission to inpatient care is necessary, this means in practice that the young person is required to eat according to a nutrition list and, if needed, to restrict exercise in order to accelerate restoration to body weight within healthy parameters. All mealtimes (six each day) are supervised by nurses and, when possible, by parents. If the young person does not eat and does not consume food by drinking high-caloric fluid (i.e., “Nutridrink”), tube feeding is advised and provided with extra kilocalories (i.e., ‘plus kcal’), if necessary by coercion (51). Zooming out more broadly, this policy—primarily oriented toward physical recovery as measured by the BMI—has been established within a biomedical positivistic context (36–39), which is grounded in a fundamental view of the human being as a rational and autonomous actor (52). Within such a view, it is argued and has been demonstrated that symptoms of EDs are worsened by severe underweight (53). Subsequently, the treatment rationale becomes to gain weight to improve the symptoms (i.e., the rational decision to take here is to stay alive and, therefore, to choose to gain weight). When the young person disagrees with this treatment rationale by refusing to gain weight, she is deemed to experience “serious disadvantage”, justifying the request for a legal authorization and start compulsory care. This illustrates how rationality is assessed as being expressed inadequately by, and posing “serious disadvantage” to, the person with severe AN; thus, conceptually, rationality and acting as an autonomous actor in severe AN are seen through a positivistic linear thinking lens as damaged, which justifies the use of compulsory care.

When, at the same time, this policy contributes to the reduction of severe underweight, it enables a transition toward a more stable and healthy state in which responsibility for eating, cessation of purging and excessive exercise, and further weight restoration can be gradually returned to the young person. The aim of this policy is not solely directed at reducing severe underweight; the aim is also to increase possibilities for and susceptibility to further treatment aimed at other themes than just the somatic parameters (e.g., number of cc tube feeding, nutrition list, number of kilocalories burned), such as the meaning of the ED and underlying problems. Put differently, to some young persons, this policy is helpful and is associated with a shift toward recovery.

#### Alternative treatment policy: Autonomy-supportive

##### Development and context of the policy

The following is based on clinical experience with providing compulsory care in youth psychiatry in The Netherlands by the treatment team (see below for a further explication of experiences). There is a group of young people for whom the treatment-as-usual (compulsory care) policy is not effective. The young person does not recover in the direction toward healthy weight parameters; the weight parameters instead become or remain seriously worrisome. Therefore, there seems to be no reason to shift responsibility back to the young person. After all, the ED dynamic has a serious grip on the young person. Paradoxically, these are often young people who have a strong need for control and autonomy and have positive ideas about the usefulness of AN in their lives. In this group, shifting responsibility to clinicians is actually associated with increased struggle and stress for the young person, making this policy ineffective or even counterproductive. Despite this policy with its coercion, a dynamic in inpatient care oftentimes evolves where the young person with severe AN continues attempting to maintain her underweight or to lose weight by exercising excessively, leaving the window open in winter, purging, hiding food at mealtimes, etc. In addition, at weighing moments, the young person can be known to hide rocks in his clothes or to drink large amounts of water to give the impression of cooperating with the treatment policy by appearing to ‘gain weight’. Evidently, this dynamic is entrenched in secrecy and distrust, leaving the young person, her parents, and the treatment team feeling lonely, worried, and powerlessness. Feelings of worry and powerlessness in the treatment team can be associated with increased coerciveness in an attempt to take care of the young person and to help her (i.e., to regain a feeling of being in control by the treatment team or to prevent death). The young person, the parents, and the treatment team are entrapped in this dynamic which can be traumatizing to all parties involved. Importantly, everyone feels pressurized in this context—the young person by her internal dynamic and by the coercion, the parents by the fear of losing their child and therefore needing the treatment team to help their child, the treatment team by feeling emotionally involved and responsible (and *being* responsible legally to provide good care which is practically usually translated as “to save a life”), and the child and youth psychiatrist who can be held legally accountable for the treatment policy and who feels personally involved as well in the suffering of the young person.

Given the details above, especially the traumatizing component for the young person, parents, and the treatment team, an alternative autonomy-supportive treatment policy was developed, which sought to tailor treatment to individual needs and as such is in accordance with an idiographic approach. Importantly, this policy did not originate from theoretical conceptualization but was developed by practice. It was informed by hands-on, bottom-up processes, continued conversations between treatment staff, critical reflection on ineffective practices, and trial-and-error learning, ultimately leading to a consensus-based and practice-oriented policy. It required a bold and courageous stance by a young, female child and youth psychiatrist (MA) holding the end-responsibility for this policy and the trust, knowledge, and developed practical skills by the coordinating psychologist (EbdV) and the treatment team (EvdB, LouiseS) in the execution of this policy. The policy was being refined each time it was executed. 

##### Content of the policy

The trusted bond, essential for a relationship in which good care can arise (54), is subject to enormous erosion due to compulsory care. Note that the initiation of compulsory care is based on the principles of effectiveness, proportionality, and subsidiarity (which can be contextualized within principlistic ethics; see the discussion for an elaboration). The principles of effectiveness and proportionality of a WvGGZ measure are thus at stake, and the termination of compulsory care seems appropriate. The goal is that the young person learns to take responsibility for healthy eating behavior in order to achieve sustainable recovery. With continued enforcement of compulsory care, there will be too little room for tapping into intrinsic motivation and the “unhealthy” situation will be maintained. The termination of compulsory care means that there are no consequences if a young person decides not to eat. The young person decides for herself what she eats. There is no nutrition list, unless the young person wants it herself, in which parents and practitioners are, of course, willing to cooperate, but the initiative lies with the young person.

This step entails an obviously high (somatic) risk of death, and this decision must therefore be taken carefully and diligently. By abolishing compulsory care and by connecting with the strong need for autonomy of the young person, no more attention and energy is spent on the battle about the nutrition list and weight. This means that the young person, on the one hand, experiences that care is provided in collaboration instead of in opposition and, on the other hand, that there is a more age-appropriate connection with the developmental tasks of the young person, such as the development of their own identity and autonomy. Of course, this policy is embedded in an understanding, caring therapeutical clinical context. In such a context, changing the treatment policy requires continuous monitoring of the efficacy of the treatment policy by empathizing with the young person, observing her, and speaking with her. Being able to detect interindividual differences as a treatment team in the response to treatment is essential in this context. The treatment team should utilize their structural diagnostic lens to assess whether this person would benefit from autonomy support and should hypothesize how the role of autonomy fits with the psychological function of the ED in its developmental and systemic context. Based on the currently limited experience with the autonomy-supportive policy, we are cautious in presenting specific diagnostic criteria guiding the clinician in indicating this policy. At the same time, the most important criterion for indicating the autonomy-supportive policy is when compulsory treatment is not effective and has observable harmful effects. More broadly, this approach fits within the ethics of care wherein people are fundamentally driven by relationality, emotionality, and mutual dependence (55–57).

Prior to this important policy change, diligent preparation is needed. This means that the young person with severe AN and parents support this policy. In addition, a second opinion has been requested to investigate whether there are other possibilities to break the impasse. A moral deliberation takes place to reflect on the ethical and moral dilemmas that this choice entails (58). Subsequently, agreements are made with the young person and parents and the pediatrician about the wishes with regard to medical intervention or treatment restrictions, such as somatic intervention in a life-threatening situation. Finally, it is important to note that the termination of compulsory care does not mean that a young person and her parents are abandoned by the treatment team. The treatment continues with a focus on collaboration and trust and is adapted to the needs of the young person and the parents.

#### Clinician-based account of experience with both policies

Below we provide a more generalized description of young people being admitted to high-intensive psychiatric care in The Netherlands in order to contextualize how the somatic, psychological, emotional, and systemic disciplines interact with each other in such cases. This general description is then refined by providing a clinician-based account of the experience of a coercion-based and autonomy-supportive policy in severe AN for two adolescents. Finally, we present the results of the analysis of the provided qualitative data of the two adolescents by sharing their experiences and reflections on the course of the treatment with us.

#### Clinical characteristics

Admitted young people (usually aged 12–18 years) are somatically in a high-risk state characterized by severe (and long-term) underweight, regular hypoglycemia, and other consequences of long-term severe underweight such as bradycardia. In the past, there are usually previous forced admissions. Psychologically, demoralization in young people and parents plays a role. In addition, a psychological conflict is usually seen wherein autonomy is shaped by determining what not to eat. Within this conflict, responsibility for the healthy side of life (e.g., going to school, maintaining friendships, forming intimate relationships, working) cannot be taken yet. The self-image is negatively colored, and coping is directed at avoiding emotions by performing perfectly. Emotionally, there are often deep feelings of loneliness and existential struggles. In terms of systemic factors, powerlessness among parents is paramount due to the intense fear of losing their child.

#### CB

##### History

2016–2020: Various hospital admissions, ED clinic, youth care, and child and adolescent psychiatry.

##### Treatment course

CB (2003) is a 17-year-old adolescent at the time of admission and with average intelligence level. CB has emotion regulation problems with ineffective coping mechanisms regarding restrictions in eating, self-mutilation, auto-intoxication, and suicidality. CB’s behavior is psychiatrically classified as personality problems and AN.

CB has had several admissions since 2016 as a result of physical deterioration by food refusal/AN. In 2016, CB was fed through a tube using coercion. CB was restrained physically by six clinicians three times a day during 3 months. CB intensely resisted the forced feeding during these months. In 2018, CB was admitted again during 10 months. Physical restraint was now applied by strapping CB onto a bed where she received forced feeding. After it was agreed to administer extra kilocalories if the food had to be administered through a tube, CB started eating independently. Contrary to the context in 2016, coercion was only applied incidentally in 2018. CB has developed traumas as a result of the coercion, for which EMDR treatment was later started. These force feeding moments have also had a major impact on her parents and on the treatment team. Treatment was continued following admission by intensive home treatment (IHT). Unfortunately, IHT was not effective as she has been admitted a number of times following IHT. The reasons for admissions in various clinics were emotion regulation problems, suicidality, and AN. In 2022, a treatment team (not the current authors) decided not to admit CB anymore to psychiatric hospitals but to guide treatment by supporting her autonomy.

#### F

##### History

2021–2022: Various hospital admissions, ED clinic, and child and adolescent psychiatry.

##### Treatment course

F (2006) has been familiar with eating problems since April 2021; she was 17 years old at the time of admission. F received forced tube feeding at a youth psychiatric hospital in the west of the country during 4 months. F showed varying physical resistance, with the frequency increasing the longer the admission lasted. After 4 months, it was decided, in consultation with Curium, F, and parents, that coerced tube feeding does not lead to the desired result, and this policy was stopped. Subsequently, F was transferred to the High and Intensive Care (H&IC) in Nijmegen with a care authorization in which it was noted that no compulsory care with regard to tube feeding had been requested. Initially, F did not manage to obtain sufficient nutrients, so she was sent to the hospital several times. Eventually, F managed to take control of her own nutritional intake, which F wanted to receive through tube feeding. F herself had the responsibility for the amount of food. The parents have stated that she can be discharged with the framework that F can be sent home if she takes in a minimum number of kilocalories per day. F built up to this lower limit and then went home with discharge, with the support of IHT where two clinicians provided systemic care with at least two home visits a week during 4 months.

These clinician-based accounts outline how a coercive framework was no longer effective in the course of long-term treatment. The applied coercion in the first account has impacted the person, her parents, and the treatment team enormously and has led to the development of the autonomy-supportive treatment policy. The second account illustrates how the autonomy-supportive treatment policy was applied in a high-risk situation.

### Part 2: Thematically ordered first-person accounts of experience with both policies

Derived from the thematic analysis, several themes are elaborated on below. The themes pertaining to the compulsory treatment policy are intrusiveness and traumatization, punishment/resistance/control, powerlessness, and professionalism. The themes pertaining to both the compulsory treatment and the autonomy-supportive policy are autonomy and control and recovery.

#### Themes pertaining to the compulsory treatment policy

1. Intrusiveness and traumatization.

The damaging, negative component of the applied coercion during treatment and its link to traumatization is clearly verbalized. It is described how the damage, which we can hypothesize to result from both physical and psychological intrusiveness by violating boundaries, impacts personal life.

“The forced treatment, the strapping up, and the physical restraint has had quite an effect on my life afterward. Many stress complaints, I have had trauma treatment for that.” (CB).

Moreover, it is described how the relational dynamic of resistance escalates in treatment with coercive elements and how this then plays out intra-individually on a psychological level. Furthermore, this alludes to the strength residing in the young person with severe AN and how this strength is directed toward not eating, not being able to cooperate with this type of treatment, and the rewarding effect of the “success” of missing food by resistance, reinforcing the resisting cycle in itself.

“Sometimes I managed to miss food with my fierce resistance, which then stimulated me to resist more. In retrospect, this resulted in traumatization.” (F).

“Moments of putting on a seatbelt in the car, getting a hug from someone [close], or being in a closed space also caused stress.”(CB).

Striking is how the intrusiveness of the coercive elements have pervasive impact on seemingly mundane day-to-day activities, such as putting on a seatbelt. The interference of the physical coercion with physical reassurance (by getting a hug) is described, where physical touch following the repeated experience of coercion during treatment is now perceived as stressful instead of soothing or helping.

The physical consequences of the applied coercion to the body are being described, again alluding to the intrusiveness of coercion. In addition, one may deem it a striking parallel that the core pathology in AN is to damage the body by restricting food intake and to witness here how the body is damaged further as a result of the coercion. The question, of course, is how the body heals from the physical damage and where scarring on the psychological and social-emotional level may remain.

“…hurt me physically. I was constantly covered in bruises and pains in my body.” (F).

2. Punishment/resistance/control.

The following statement describes how forced feeding feels like being punished for something one is incapable of stopping, halting, or mitigating herself as the process of refusing food is being described as a disease and contributes to a dynamic where there is resistance between the youth and the clinicians. One can imagine how such a repeated process would contribute to an erosion of the feeling of trust in the caretaking process.

“The extra calories that were added as soon as the feeding had to be given through the tube felt like being punished extra while it was the disease that made me refuse food. During tube feeding, this resulted in extra resistance.” (CB).

Here a youth describes how the internal anorectic process of punishing oneself by being resistant becomes intertwined and entangled with the forced feeding moments during treatment. The role of feelings of guilt in maintaining the anorectic process is elucidated clearly here.

“In my head I thought if I did get food, which was often the case, at least I wouldn’t have cooperated. Then, I would have tried it anyway [to resist] and at least I felt a little less guilty toward my ED.” (F).

Here it is verbalized how punishment is incorporated in the treatment process by isolating the youth for a longer period of time. This also alludes to how eating and drinking are weaponized during the treatment processes—while this may not be the intention. A parallel can be observed again between the internal anorectic process and the processes unfolding during treatment in an inpatient setting.

“There was the rule, if you don’t eat then you go into the white room, until you eat. I stopped eating and drinking, which resulted in being locked up in the white room and after three days I was transferred.” (CB).

3. Powerlessness.

A youth describes here how years of treatment attempts have been unsuccessful in recovering from her ED and how this resulted in feelings of powerlessness of everyone involved.

“After the compulsory treatment, many more admissions to other institutions followed, but without real improvement, which eventually resulted in a situation where everyone had their backs against the wall.” (CB).

4. Professionalism.

The importance of professional training for nurses in how to act during moments of coerced feeding and the difference this makes in being physically hurt is depicted here.

“I have seen the difference in clinicians that were and were not trained for it. That is why I think it is important that clinicians know how to act. The coercion by nurses who didn’t know how to deal with such situations hurt me physically.” (F).

Here how feelings of frustration by clinicians may become interwoven into the process of applying coercion and are being felt by youth with severe AN during coerced feeding are being referred to. The importance of clinical intervision and supervision procedures is implicitly mentioned by this youth, as these procedures are known to alleviate feelings of frustration in how to handle high-stake situations, such as forced feeding moments. There is also a description of how being restrained on a bed is at the same time psychologically bizarre and also being perceived as safe.

“I think it remains very important that clinicians realize that the young people themselves are not the ED and therefore must not take out their frustrations on the person herself. At the H&IC, they could deal with this. The idea of being tied up on a bed is bizarre, but it is quieter and safer for everyone involved.” (F).

Here how youth with severe AN can be admitted to youth care facilities—where caretaking is perceived as different, of lesser quality, or even more harmful compared to caretaking in psychiatric clinics—is being referred to. Note how a distrusting culture is evident in how youth are relationally treated and how basic tenets of treatment processes (i.e., building a trusting relationship) are being violated.

“The guidance there was different from psychiatric clinics. Staff were unkind, used bad language, physical violence that you saw happening, police crossing the line. Upon entering you were searched, possibly wearing a torn dress and in a kind of isolation cell which was a white room without stuff and with tinted windows.” (CB).

The lack of providing information on the possible harmful, traumatizing effects of compulsory care and how self-blame and self-stigma interferes with seeking help for traumatic experiences is described here. As such, this statement also underscores the importance of actively informing the youth about trauma and promoting help-seeking behaviors by clinicians involved in the coercive elements of treatment.

“The thoughts such as ‘it must be part of it, I’m exaggerating’ made me not dare to take the step to trauma therapy.’” (CB).

Given the intense experience during the treatment process of severe AN both to the youth and the clinicians involved, there is a need voiced below for follow-up contacts to exchange experiences. In clinics where compulsory care takes place, this is not an integral part of treatment currently, to the best of our knowledge.

“There are things to be gained in terms of treatment afterwards: follow-up in terms of trauma and conversations with those who gave it. It is intense for both parties to cross someone’s boundary like this, so it makes sense that it does a lot [has major impact] for both parties.” (CB).

#### Themes pertaining to the compulsory treatment policy and autonomy-supportive policy

1. Autonomy/control.

Acknowledging the feelings of powerlessness and facing the reality that there had been no real improvement set the stage for treatment wherein autonomy was the guiding principle in one of the youths.

“Following the period where my BMI was dangerously low, all care was then geared to the fact that it was up to me (autonomy support). It was my choice to eat again or not. I was no longer hospitalized, no matter how bad things were.” (CB).

This statement alludes to how it is a dilemma for everyone involved in decision-making and providing treatment for youth with severe AN as she describes how she feels she was unable to make healthy choices herself.

“I wasn’t exactly able to make healthy choices about my own treatment [at the time of admission].” (F).

2. Recovery.

The importance of feeling part of society to the process of recovery is highlighted here, as it is described here how studying and working as a volunteer help in the broadening of the identity as not being solely a patient but also having value as a person contributing to society. The engulfing experience of having an ED is also implicitly mentioned—given how she has been unable during the time of having an ED to take on a role outside that of the patient.

“I am good. Slowly, I am shaping my life again. The ED is under control. I now live permanently with my mother. I have completed a self-study and I work as a volunteer at the food bank and at UNICEF. It is nice to get out of the role of patient; that is valuable.” (CB).

The life-saving element of coerced feeding during treatment is mentioned here. One could argue that, as such, coerced feeding may also set the stage for recovery processes.

“It’s what kept me alive, and I’m happy with that in retrospect.” (F).

## Discussion

In part one of our study, we have described what a compulsory treatment policy in the Dutch context entails and an autonomy-supportive alternative to such a policy when compulsory care is not effective. By presenting two case studies, we have illustrated what these policies mean to youth receiving the policies. Furthermore, the results from our second part of the qualitative study indicate that the youths experience points to intrusiveness, traumatization, and resistance regarding the coercive elements within compulsory care. For the autonomy-supportive policy, intrusiveness, traumatization, and resistance are not named by the youths’ reflection on this treatment policy. For both of the compulsory care and the autonomy-supportive treatment policy, there are also reflections on recovery and the role of autonomy and control. Finally, recommendations on how to professionalize compulsory care, if any, are given by youths with severe AN.

In the case where compulsory treatment is not successful—where deterioration of symptoms and inflicted harm are being observed despite compulsory treatment—we have described an alternative following compulsory care, that being an autonomy-supportive treatment policy. When compulsory care for adolescents with severe AN proves ineffective, we propose an autonomy-supportive treatment policy that offers greater space, attention, and care for the person beyond AN. This approach also strengthens the therapeutic relationship (54, 59, 60) by emphasizing collaboration rather than coercion. The policy is theoretically grounded in positivism (36–38, 61) and social constructivism (4, 43, 45), acknowledging the interplay of somatic, emotional, psychological, and systemic factors. Moreover, the ethics of care—highlighting relationality, emotionality, and mutual dependence as central to human experience (55–57)—guide the ongoing refinement and implementation of this autonomy-supportive alternative.

At the highest levels of escalation (cf. 14), emphasizing relationality and adopting an autonomy-supportive treatment policy may function as a “parachute” that enables stepping down the escalation ladder. The core principles of care ethics clarify this metaphor. A strong, collaborative *relationship* provides the trust needed to take such a leap. The parachute symbolizes the capacity for adolescents with severe AN, their parents, and the treatment team to explore unfamiliar terrain and gain shifting perspectives, while its steering reflects the centrality of autonomy. Direction, however, depends on staying connected to *emotions*, which act as a compass. Mid-air, adolescents, parents, and clinicians must remain in close proximity to observe, communicate, and support one another, underscoring *mutual dependence*. These tenets align with the psychiatric recovery movement’s emphasis on meaningful life (62) and with the principles of new authority in parental guidance for persistent psychiatric problems (63).

The two stories presented reveal challenges across the disciplines involved. Philosophically, reliance on a principlist ethical framework (52) is limited in high-stake AN care. Under this model, clinicians—typically psychiatrists—may judge that AN overrides the individual’s autonomy and rationality, thereby justifying compulsory care (64). Such assessments of impaired autonomy in adolescents with severe AN can be understood as forms of epistemic injustice (65, 66), as they undermine these adolescents’ capacity to make sense of their own experiences. Indeed significant aspects of their perspectives are disregarded in decisions to initiate compulsory care, and both individuals reported lasting negative effects of coercion. Failing to adequately hear their experiences not only restricts their epistemic agency but also inflicts harm.

Integrating biomedical ethics with the ethics of care places relationality at the center, fostering collaboration and validating the adolescents’ emotional experiences. Broadening the theoretical lens also encourages a shift in language: moving away from pseudo-neutral yet stigmatizing psychiatric terminology (67) toward a lexicon that recognizes emotional pain not as something to be suppressed but as meaningful and potentially transformative. Clinicians can support adolescents with severe AN by offering affirmative recognition (68)—acknowledging their suffering—and then providing transformative recognition, which reframes emotional pain as part of life and opens the possibility of recovery, consistent with concepts such as emodiversity (69).

From a societal perspective, implementing the termination of compulsory care requires acknowledging AN as a “wicked problem” (35) as well as considerable courage from adolescents, parents, and treatment teams (70) and acceptance of the risks of potentially irreversible organ damage and even death. Nevertheless, we argue that this policy becomes the only remaining option when (a) a young person’s drive for autonomy—however shaped by the ED—functions as a destructive, self-perpetuating pattern and (b) the developmental task of autonomy is further undermined by invasive compulsory interventions. In this context, the risk of iatrogenic harm from compulsory care no longer applies, allowing clinicians to adhere to the ethical principle of *primum non nocere* (52).

The adolescents’ accounts align with prior qualitative findings (2) that young people with severe AN often feel unheard within compulsory care and experience it as traumatic, particularly when physical restraint is used (CB) or applied by untrained staff (F). Consistent with earlier research (2), both adolescents acknowledged the life-saving importance of weight restoration but viewed a predominantly physical focus in treatment as insufficient. Previous studies also highlight the value of connecting with the person beyond AN and maintaining hope (41), although these themes did not emerge explicitly in our two cases. Similarly, while focusing on personal values can support recovery (42), this was not affirmed in our data; one adolescent instead emphasized that support for autonomy was most helpful. When practitioners themselves feel disconnected from their values—particularly when coercive compulsory care is experienced as traumatizing for both adolescent and clinician—it becomes exceedingly difficult for them to guide young people through the inherently ambivalent recovery process (25, 26, 29).

This study is limited by the small number of case reports and by its grounding in the Dutch context, which restricts generalizability given the strong cultural dependency of compulsory treatment practices. In addition, the authors’ prior professional collaboration at a youth psychiatric crisis center providing inpatient compulsory care (except for CV and CB) may have shaped a favorable orientation toward the autonomy-supportive policy. Replication in other settings and countries and by independent clinical teams is therefore necessary to assess the robustness of these findings. Despite these limitations, the study offers insight into a typically closed clinical environment and foregrounds youths’ perspectives through detailed case reports.

### Research implications

Future research should place greater emphasis on qualitative approaches that center the experiences of young people and practitioners to improve care for adolescents with severe AN, particularly within compulsory treatment, with attention to relational and value-driven practices. Studies examining the experiential impact of how compulsory care is delivered—when it is used—are especially needed. Given the scarcity of quantitative research and the limitations of conventional efficacy designs such as randomized controlled trials for this population, single-case designs and idiographic quantitative methods, including ecological momentary assessment, are warranted (5). Conceptually, broader dialogue among adolescents, parents, and clinicians about the dilemmas and considerations involved in compulsory care is essential. Finally, it is timely to reflect on and update the foundational conceptual assumptions underlying current treatment models in light of contemporary knowledge and experience (e.g., 71).

### Clinical and policy implications

Caring for young people who refuse food and face a life-threatening risk naturally evokes powerlessness, frustration, and worry among clinicians. Institutional leaders should therefore ensure that intervision, supervision, and structured follow-up are integral components of clinical practice so that caregivers themselves feel supported (72). Importantly, both of the young people described in this study are alive today, and one indicated that post-treatment follow-up meetings with the team would have been helpful. Similar needs have been reported by clinicians in focus groups on the impact of forced feeding: they expressed a strong desire to know how adolescents treated with coercion fare afterward, including through follow-up meetings (72). Such follow-up processes should be standard within psychiatric institutions.

Those responsible for funding care must also recognize the intense emotional and relational demands of inpatient treatment and allocate sufficient resources to support the caregiving process. Clinicians, in turn, must engage in ongoing reflection on the emotions that arise during treatment to prevent policies from being shaped by unprocessed feelings of powerlessness, anger, or fear—which can lead to controlling practices (73). Treatment should instead be grounded in the capacity to hold ambivalence and to act from trust and hope (cf. 74). After all, as Gregory Bateson described: “The pathology is to want control, not that you ever get it, because of course you never do.”

## Data Availability

The datasets presented in this article are not readily available because it contains sensitive information. Requests to access the datasets should be directed to l.l.stone@tilburguniversity.edu.

## Footnotes

1 In The Netherlands, adolescents aged 12–16 are subject to parental consent. From 16 years onwards, they consent independently.
